# Urinary and sexual outcomes in long-term (5+ years) prostate cancer disease free survivors after radical prostatectomy

**DOI:** 10.1186/1477-7525-7-94

**Published:** 2009-11-13

**Authors:** Mauro Gacci, Alchiede Simonato, Lorenzo Masieri, John L Gore, Michele Lanciotti, Annalisa Mantella, Mario Alberto Rossetti, Sergio Serni, Virginia Varca, Andrea Romagnoli, Carlo Ambruosi, Fabio Venzano, Marco Esposito, Tomaso Montanaro, Giorgio Carmignani, Marco Carini

**Affiliations:** 1 Department of Urology, University of Florence, Careggi Hospital, Florence, Italy; 2 "L. Giuliani" Department of Urology, University of Genoa, Genoa, Italy; 3 Department of Urology, David Geffen School of Medicine at UCLA, Los Angeles, CA, USA

## Abstract

**Background:**

After long term disease free follow up (FUp) patients reconsider quality of life (QOL) outcomes. Aim of this study is assess QoL in prostate cancer patients who are disease-free at least 5 years after radical prostatectomy (RP).

**Methods:**

367 patients treated with RP for clinically localized pCa, without biochemical failure (PSA ≤ 0.2 ng/mL) at the follow up ≥ 5 years were recruited.

Urinary (UF) and Sexual Function (SF), Urinary (UB) and Sexual Bother (SB) were assessed by using UCLA-PCI questionnaire. UF, UB, SF and SB were analyzed according to: treatment timing *(age at time of RP, FUp duration, age at time of FUp)*, tumor characteristics *(preoperative PSA, TNM stage, pathological Gleason score)*, nerve sparing (NS) procedure, and hormonal treatment (HT).

We calculated the differences between 93 NS-RP without HT (group A) and 274 non-NS-RP or NS-RP with HT (group B). We evaluated the correlation between function and bother in group A according to follow-up duration.

**Results:**

Time since prostatectomy had a negative effect on SF and a positive effect SB (both p < 0.001). Elderly men at follow up experienced worse UF and SF (p = 0.02 and p < 0.001) and better SB (p < 0.001).

Higher stage PCa negatively affected UB, SF, and SB (all: p ≤ 0.05). NS was associated with better UB, SF and SB (all: p ≤ 0.05); conversely, HT was associated with worse UF, SF and SB (all: p ≤ 0.05).

More than 8 years after prostatectomy SF of group A and B were similar. Group A subjects (NS-RP without HT) demonstrated worsening SF, but improved SB, suggesting dissociation of the correlation between SF and SB over time.

**Conclusion:**

Older age at follow up and higher pathological stage were associated with worse QoL outcomes after RP. The direct correlation between UF and age at follow up, with no correlation between UF and age at time of RP suggests that other issues (i.e: vascular or neurogenic disorders), subsequent to RP, are determinant on urinary incontinence. After NS-RP without HT the correlation between SF and SB is maintained for 7 years, after which function and bother appear to have divergent trajectories.

## Background

Prostate cancer and its treatments are costly and significantly impact quantity and quality of life; moreover, most prostate cancer survivors receive a significant portion of their care as outpatients [1]. Radical prostatectomy, in addition to represent one of the best approach for long term cancer control in clinically localized PCa [2], has a remarkable impact on patient's quality of life (QoL). Although the primary goal of any innovative treatment for prostate cancer is to maximize life expectancy, both patients and clinicians are currently devoting more attention to the impact of current therapies on QoL outcomes [3]. For many patients, the QoL impact of treatment determines the therapy selection among the currently available approaches [4]. Toward that end, several new surgical developments have attempted to maximize QoL after prostatectomy [5].

Urinary incontinence and erectile dysfunction are the most prominent side effects of radical prostatectomy [6]. The severity of patient-reported symptoms can be very different from symptom-related bother. Several items can affect both symptoms and bother in different ways. Urinary and sexual symptoms and bother are usually dependent on age at the time of surgery [7]. In addition, after long-term disease-free follow-up, patients have a propensity to reconsider their QoL status [8], even if aging can worsen overall patient health. Moreover, patients with high-risk PCa may better tolerate long term adverse events than those with low-risk PCa. Finally, a bilateral nerve-sparing approach, as well as the requirement for postoperative hormone treatment, can be major determinants of sexual QoL after prostatectomy [9].

The aims of the present study are: 1) to assess QoL outcomes in prostate cancer survivors who are disease-free at least 5 years after radical prostatectomy, 2) to identify the primary determinants of long-term QoL, and 3) to evaluate the impact of nerve-sparing surgery without hormone therapy on long-term urinary and sexual outcomes.

## Methods

### Study population

Our study population was composed of patients who had undergone radical retropubic prostatectomy (RP) for PCa in 2 centers of excellence between 1995-2002. Patients included underwent RP with either a bilateral nerve (NS) or non-nerve sparing (non-NS) approach as primary therapy for clinically localized prostate cancer (cT1-cT2, N0, M0), maintained a postoperative PSA ≤ 0.2 ng/mL with follow-up of at least 5 years, and completed our study questionnaire. The follow-up schedule included serum PSA assay every 3 months for the first year, then every 6 months for the following two years and yearly thereafter. Biochemical relapse was defined as evidence of PSA > 0.2 ng/mL at two consecutive measurements.

Informed consent was obtained from all subjects. This trial was carried out in accordance with the ethic principles of the Helsinki declaration (1996) and good clinical practice issues (1997) and was reviewed and approved by both the local ethics committee.

We excluded those with preoperative urinary incontinence (assessed by medical history, at time of hospitalization), those who received neoadjuvant or adjuvant radiotherapy, those with incomplete pre or postoperative data, those who underwent unilateral NS-RP or in whom the NS status could not be determined, with an inability to complete the questionnaire, and refusal to participate. Furthermore, patients without a partner or without a sexual activity in the year before prostatectomy were excluded from the study, to improve the assessment of the sexual bother outcomes.

Patients with preoperative PSA ≤ 10 ng/ml, biopsy Gleason score ≤ 7, age at diagnosis ≤ 70 years, and preoperative IIEF score ≥ 20 were selected for NS-RP. All patients treated with a NS-RP used several ED treatments (including vacuum device, penile injections, and recently PDE-5 inhibitors), used subsequently or in combined therapies, with the aim to preserve or recover their sexual function.

All RP specimens were fixed in formalin, coated with India ink, weighed, and serially sectioned, staged, and graded according to the 2002 American Joint Committee on Cancer (AJCC) staging system.

Follow-up included serum PSA every 3 months for the first two years, every 6 months for the following three years, and yearly thereafter. Biochemical relapse was defined as PSA > 0.2 ng/ml on 2 consecutive measurements. Patients with biochemical recurrence were treated with adjuvant hormonal therapy (LHRH analog with or without anti-androgen) at time of biochemical relapse.

### HRQOL measures

We used the validated Italian version of the UCLA Prostate Cancer Index (PCI) [10], that assesses urinary continence and sexual function and their impact on related bother. We directly interviewed patients face to face, and they completed the questionnaire in a self reported fashion. This questionnaire allows evaluation of the detailed symptoms as well as their corresponding bother. For this analysis, we focused on subject urinary and sexual function (UF and SF) and urinary and sexual bother (UB and SB). Responses were scored from 0 to 100, with a higher score indicating better QoL.

### Statistical analysis

We evaluated in the statistical analysis the correlation between function and bother and subject demographic and clinical characteristics with Pearson correlation coefficients. Variables that were significant on univariate analysis were incorporated into a linear regression model (forward, stepwise variable entry) for multivariate analysis of factors influencing the items to evaluate postoperative urinary and sexual QoL over time. SF and SB outcomes were evaluated for all patients (n = 367) and for those who underwent NS-RP without HT (Group A, n = 93). Differences at 4 follow-up times (5, 6-7, 8-9, and ≥ 10 years) in UF, UB, SF and SB scores between Group A subjects and the other 274 subjects treated with non-NS-RP or NS-RP and subsequent HT (Group B) were assessed by using unpaired samples t-tests. Finally, we calculated the correlation between UF and UB and between SF and SB of subjects in Group A at the above mentioned 4 different follow-up intervals with Pearson correlation coefficients.

## Results

### Patient characteristics

We overall collected 367 questionnaires: 307 men presented a follow up time > 5 years (mean 95.5 months, r: 61-156), while for the remaining 60 men follow up time was 5 years (60 months). Clinical presentation, pathological findings and follow up time of the whole population and both subgroups of 5 years amd more than 5 years follow up are listed in table 1. Mean age at RP was 64.8 years (median 66, range 47-77) and mean follow-up time was 89.7 months (median 84, range 60-156). Sixty subjects (16.3%) had follow-up of 5 years, 146 subjects (39.8%) had 6-7 years follow-up, 81 subjects (22.1%) had 8-9 years follow-up, and 80 subjects (21.8%) had follow-up beyond 10 years. Of the 367 subjects, mean preoperative PSA was 14.6 ng/ml (median 10.2, range 0.8-87): 165 (45.0%) had a PSA <10, 134 (36.5%) had a PSA between 10-20, and 68 (18.5%) had a PSA > 20 ng/ml. Pathologic stage was T2 in 222 subjects (60.5%), pT3a in 77 subjects (21.0%), pT3b in 59 subjects (16.1%) and pT4 in 9 subjects (2.4%). Median pathological Gleason score was 7: ≤ 6 in 154 subjects (42.0%), 7 in 146 subjects (39.8%), and 8-10 in 67 subjects (18.2%).

**Table 1:** Clinical presentation and pathological findings of the 367 patients

	Overall	F.up 5 yy	F.up > 5 yy
***N° of patients***	367	60	307
***Mean age (years), (median, range)***	64.8 (66, 47-77)	64.1 (65, 49-74)	64.9 (66, 47-77)
***Mean follow up (months), (median, range)***	89.7 (84, 60-156)	60	95.5 (87, 61-156)
***Follow up time: (years) n (%)***			
5		60 (16.3)	
6-7			146 (39.8)
8-9			81 (22.1)
> 10			80 (21.8)
***Pre-operative PSA (ng/ml)*** ***mean (median, range)***	14.6 (10.2, 0.8-87)	12.8 (9, 3.9 - 63)	15 (10.6, 0.8-87)
***Pre-operative PSA (ng/ml)***	***n (%)***	***n (%)***	***n (%)***
<10	165 (45.0)	34 (56.6)	131 (42.7)
10-20	134 (36.5)	17 (28.3)	121 (39.4)
>20	68 (18.5)	9 (15.1)	55 (17.9)
***Specimen Gleason Score***	***n (%)***	***N (%)***	***n (%)***
2-6	154 (42.0)	31 (51.7)	123 (40.1)
7	146 (39.8)	19 (31.7)	127 (41.3)
8-10	67 (18.2)	10 (16.6)	57 (18.6)
***Pathological stage (TNM 1997)***	***n (%)***	***n (%)***	***n (%)***
*T2*	222 (60.5)	40 (66.7)	182 (59.3)
*T3a*	77 (21.0)	13 (21.6)	64 (20.8)
*T3b*	59 (16.1)	6 (10)	53 (17.3)
*T4*	9 (2.4)	1 (1.7)	8 (2.6)
***Nerve sparing***	***n (%)***	***n (%)***	***n (%)***
	125	24 (19.2)	101 (80.8)

NS-RP was performed in 125 subject (34.1%): 24/60 (40%) patients with a follow up time of 5 years, and 101/307 (33%) with a follow up time >5 years (see table 1), The remaining 242 subjects (65.9%) underwent non-NS-RP. Only recently (in the last 5 years) we used structured procedures, with inclusion/exclusion criteria and scheduled treatment protocols either of profilaxis and treatment of post prostatectomy ED. All patients with a follow up time > 5 years did not undergo a structured rehabilitation protocol for ED: the starting timing of drugs administration was not the same, and in many cases men started ED treatment several months after surgery. Furthermore the treatment of ED was outlined with different devices (PDE5, PgE, vacuum), used subsequently or in combined therapies. On the contrary, the remaining 24 patients with a follow up time of 5 years (19.2%) undergone a structured profilaxis for postprostatectomy ED [11]: at follow up time 10 patients were using PDE5-i, 9 PDE5-i plus PGE, 1 patient needed the use of a vacuum device and 4 patients did not use any aids at all. For the heterogeneous data from patients with follow up >5 years and the small population of men with 5 years follow up we avoided the stratified analyses according to the use of erectile aids.

Seventy-six subjects (20.7%) received adjuvant HT. Overall, 93 patients (25.3%, Group A) treated with NS-RP did not require HT, while 274 (74.7%, Group B) underwent either non-NS-RP (242 patients, 88.3%) or NS followed by HT (32 patients, 11.7%).

### Univariate analysis

On univariate analysis (Table 2), urinary function was worse in older subjects, with adverse tumor characteristics and hormone treatment likewise correlated with worse continence. Subjects with unfavorable tumor characteristics and under HT also reported worse UB scores, while those treated with NS-RP endorsed better UB compared with those undergoing non-NS surgery.

**Table 2:** Univariate analysis of the whole study sample with Pearson correlation coefficients

	Timing			Tumor characteristics			Nerve-sparing	Hormone therapy
			
Pearson r p-value	Age at RP	Follow-up duration	Age at follow-up	PSA	T-stage	Gleason score		
**UF**	-0.093 0.075	-0.091 0.080	**-0.119** **0.023**	**-0.167** **0.001**	**-0.139** **0.008**	**-0.184** **0.001**	0.096 0.066	**-0.131** **0.017**

**UB**	-0.049 0.348	-0.079 0.131	-0.076 0.145	**-0.142** **0.006**	**-0.163** **0.002**	**-0.156** **0.004**	**0.117** **0.025**	-0.105 0.055

**SF**	**-0.247** **< 0.001**	**-0.214** **<0.001**	**-0.298** **<0.001**	**-0.111** **0.033**	**-0.144** **0.006**	**-0.150** **0.006**	**0.272** **<0.001**	**-0.113** **0.039**

**SB**	0.061 0.244	**0.240** **<0.001**	**0.144** **0.006**	0.050 0.338	**-0.180** **<0.001**	-0.033 0.552	**0.162** **0.002**	**-0.143** **0.009**

Significant correlations (p ≤ 0.05) are in bold.

[UF: urinary function; UB: urinary bother; SF: sexual function; SB: sexual bother; RP: radical prostatectomy]

Treatment timing, tumor characteristics, and HT were all negatively correlated with SF on univariate analysis, while NS-RP was positively correlated with SF. Those with longer follow-up, older age at follow-up, and those treated with NS-RP had less sexual bother. Subject with higher pathological stage and those who received HT had worse SB.

### Multivariate analysis

Multivariate analysis (Table 3) showed a significant positive correlation between follow-up duration and SB and an inverse correlation between age at follow-up and UF. Moreover, pathological stage negatively affected UB, SF, and SB. Multivariate analysis confirmed the positive effect of NS on UB and SB and corroborated the negative effect of HT on UF.

**Table 3:** Multivariate analysis of the whole study sample with logistic regression model (forward, stepwise variable entry)

	Timing			Tumor characteristics			Nerve-sparing	Hormone therapy
			
r p-value	Age at RP	Follow-up duration	Age at follow-up	PSA	T-stage	Gleason score		
**UF**	/	/	**-0.609** **0.024**	-3.557 0.163	-3.103 0.163	-2.878 0.072	/	**-5.607** **0.056**

**UB**	/	/	/	-2.944 0.207	**-4.161** **0.051**	-2.272 0.125	**2.774** **0.046**	-3.718 0.189

**SF**	-1.304 0.617	-0.229 0.302	0.176 0.946	-1.104 0.601	**-4.264** **0.024**	-2.106 0.110	**3.783** **0.004**	**-4.259** **0.044**

**SB**	/	**0.290** **<0.001**	0.272 0.336	/	**-4.852** **0.018**	/	**5.101** **<0.001**	-7.205 0.076

Significant results (p ≤ 0.05) are in bold.

[UF: urinary function; UB: urinary bother; SF: sexual function; SB: sexual bother; RP: radical prostatectomy. [/: Not included for the multivariate analyses]]

### Sexual function and sexual bother after NS-RP without HT

Concerning sexual function and bother after NS-RP without hormonal treatment, sexual bother was not influenced by timing or tumor characteristics (Table 4). On the contrary, age at follow-up and pathological stage negatively affected SF on both univariate and multivariate analysis.

**Table 4:** Univariate and multivariate analyses of subjects treated with nerve-sparing RP without hormone treatment.

	Timing			Tumor characteristics		
	
r p-value	Age at RP	Follow-up duration	Age at follow-up	PSA	T-stage	Gleason score
SF*	-0.204 0.050	-0.184 0.077	**-0.258** **0.012**	0.053 0.612	**-0.207** **0.046**	-0.096 0.359

SB*	0.098 0.350	0.161 0.124	0.143 0.170	-0.064 0.543	-0.015 0.889	-0.031 0.771

**SF#**	1.841 0.303	/	-3.138 0.056	/	**-12.530** **0.027**	**/**

Significant results (p ≤ 0.05) are in bold.

[SF: sexual function; SB: sexual bother./: Not included for the multivariate analyses]

### Differences between NS without HT and non-NS or NS with HT at interval follow-up

**Figure 1:**
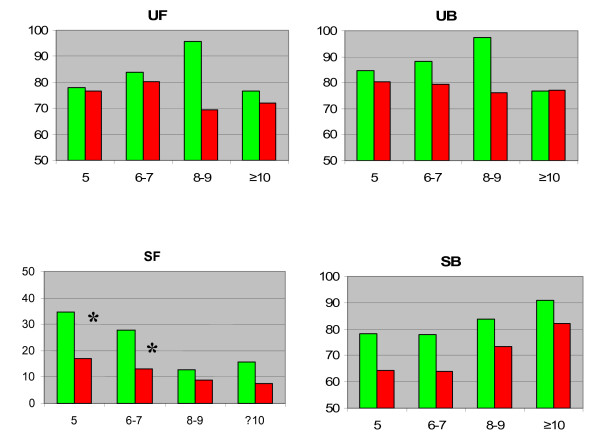
**Mean UF, UB, SF, and SB scores in subjects who underwent NS-RP without HT (Group A - green bars) and non-NS-RP or NS-RP with HT subjects (Group B - red bars), stratified according to years of follow up**. UF: urinary function; UB: urinary bother; SF: sexual function; SB: sexual bother. [* p < 0.05].

We analyzed differences between patients who underwent a nerve sparing procedure without HT and non NS or NS with HT at 4 different follow up intervals. No differences in UF, UB and SB were noted between Group A and Group B subjects. As expected, patients treated with bilateral nerve sparing prostatectomy, without hormone presented better SF and SB scores in each time point (**SF**: NS without HT: 5 yy: 34,61, 6-7 yy: 27,78, 8-9 yy: 12,60, ≥ 10 yy: 15,77. NNS or NS with HT: 5 yy: 17,06, 6-7 yy: 12,97, 8-9 yy: 8,71, ≥ 10 yy: 7,42. **SB**: NS without HT: 5 yy 78,26, 6-7 yy: 77,78 yy, 8-9 yy: 83,75, ≥ 10 yy: 91,07. NNS or NS with HT: 5 yy 64,19, 6-7 yy: 63,86, 8-9 yy: 73,36, ≥ 10 yy: 82,20). Furthermore, subjects who underwent NS-RP without HT reported significantly higher SF scores 5-7 years postoperatively compared with Group B subjects [See Figure 1].

The high urinary function and bother scores in the NS without HT group at follow up 8-9 years (see Figure 1), can be explained by the low number of patients (20), with the consequent lack of worse urinary outcomes.

### Correlation between symptoms and bother at interval follow- up in Group A subjects

**Figure 2:**
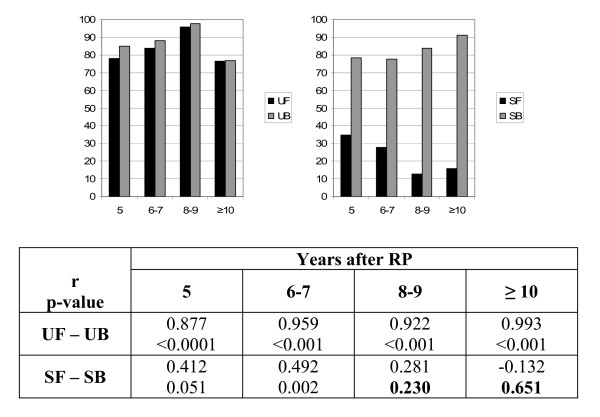
**Comparison of function and bother among Group A subjects at interval follow-up**. The table reports Pearson correlation coefficients and p-values assessing the correlation between function and bother. Non-significant results (p > 0.05) are in bold. UF: urinary function; UB: urinary bother; SF: sexual function; SB: sexual bother; RP: radical prostatectomy.

For the analysis of correlation between symptoms and bother at 4 different follow up intervals in Group A subjects, as shown in Figure 2, our subjects reported similar UF and UB scores. Moreover, correlation coefficients between UF and UB scores were very similar at each of the 4 follow-up intervals. Increased follow-up duration was characterized by a progressive deterioration in SF and an improvement in SB, with the consequent dissociation of the correlation between SF and SB from 8 to 10 years after RP.

## Discussion

Urinary and sexual outcomes following RP may differ by age: younger men usually have better preservation of urinary and sexual function after RP, with less bother compared to older men [12]. Higher baseline urinary and sexual function scores among younger men may drive their superior age-related outcomes [13]. Furthermore, urinary and sexual function usually worsens with age [14]: in a population-based study on long-term prostate cancer survivors, urinary incontinence and erectile dysfunction occurred more often among post-prostatectomy patients compared with the regular population, differences that cannot be explained merely by age [15]. In our study, worse UF was most common in older men several years after surgery. In particular, age at follow-up had the strongest association with postoperative incontinence, all other covariates held constant. In addition, after NS-RP without HT, follow-up age was negatively associated with erectile function: these older men reported erectile dysfunction independent of age at the time of RP and follow-up duration. Patients selected for NS-RP who did not require subsequent HT were essentially cancer-free; age at follow-up was the foremost determinant for sexual function. Among the entire cohort, longer disease-free interval after surgery seems to be associated with reduced sexual bother independent of patient age. The absence of biochemical failure several years after RP may allow better tolerance of postoperative erectile dysfunction.

RP in locally advanced prostate cancer (pT3) offers the potential for cancer control with or without additional treatment [16]. In a retrospective study on RP performed in high risk prostate cancer, Catalona reported a preservation of continence and potency in 92% and 64% of cases respectively [17]. Furthermore, Zincke confirmed the good disease-free outcomes in long-term follow-up, and reported a complication rate in T3 patients similar to that among patients with T2 Pca [18]. The Department of Urology of the University of Florence is a centre of excellence for advanced (pT3) prostate cancer [19,20]. This can explain the high rate of pT3 patients and the low rate of nerve sparing procedure of the whole population, compared to other centers experience. In our study, we confirmed a similar continence rate between low and high stage PCa patients. Moreover, more advanced stage was associated with worse sexual outcomes. Among our entire cohort, this seems to be due to the selection of the majority of these patients for non-NS-RP in those with cT3 Pca. The negative association between stage and sexual function among Group A patients (NS-RP without HT) may relate to more difficult dissection of the neurovascular bundle from the prostate capsule and the avoidance of an intrafascial dissection of the periprostatic neurovascular bundle.

Consistent with analyses of QoL outcomes among RP patients, the potency rates reported herein after NS-RP without HT were associated with reduced sexual bother. The sexual function outcomes among this group were superior to those undergoing non-NS-RP and NS-RP with HT up to 7 years after surgery (see figure 1, panel SF). Beyond 7 years postoperatively, age-related erectile dysfunction may explain the equilibration of sexual function outcomes between these two patient groups. Moreover, our population resulted in general less bothered than other populations such as reported in literature [21]. This data can be easily explain by the remarkable impact on the disease free status (PSA < 0.2 ng/mL) at long term follow up time (> 5 years) of our patients: the conviction of an effective cancer control allow a better acceptance of sexual comorbidities.

We found a non-significant trend toward better continence after NS-RP. Several authors have reported that NS confers improved postoperative urinary continence [22,23]. The lack of a correlation in our series may be related to the advanced age at follow-up and the consequent age-related incontinence of our cohort. Interestingly, men treated with NS-RP had significantly less urinary bother compared with men who underwent non-NS-RP, independent of the degree of urinary incontinence. Urinary bother may correlate more closely with the severity of storage urinary symptoms, more common after external beam radiation therapy or brachytherapy, rather than with the degree of urinary incontinence [24].

Finally, the analyses of Group A men stratified by follow-up duration demonstrated that, while minimal urinary symptoms were associated with increased distress related to those symptoms, the progressive development of erectile dysfunction is well tolerated 8 or more recurrence-free years after surgery. This confirms that minimal urinary incontinence continues to be poorly tolerated even after several years of good cancer control, while erectile dysfunction progressively diminishes as a problem in the daily life of long-term disease-free survivors.

Our study presents several limitations. First of all, we did not include some factors that may have biased our outcomes, such as marital status, education level, employment status, and income. We were, however, able to account for factors known to have a substantial influence on postoperative QoL, such as patient age, pathological features of the PCa, NS status, and the administration of hormone therapy. Moreover, all recruited men underwent RP in centers of excellence by skilled urologists. Thus, our patient population and QoL outcomes may be not representative of the general population. Furthermore, we did not evaluate generic and general oncological QoL with validated instruments such as the Medical Outcomes Study Short Form-12 or the European Organization for Research and Treatment of Cancer QOL-30. Finally, our findings have the inherent limitations of a retrospective study, most prominently a lack of baseline QoL data.

## Conclusion

We demonstrated that long-term RP outcomes follow a distinct QoL trajectory. Older men develop worse urinary continence independent of age at time of surgery or follow-up duration. Pathological stage was an important determinant of postoperative QoL outcomes, affecting both urinary and sexual function. Beyond 8 years after NS-RP without HT, patients noted substantial sexual dysfunction, but, surprisingly, they were minimally sexually bothered. These results contribute to the clinician's ability to counsel long-term prostate cancer survivors.

## Abbreviations

RP: Radical Prostatectomy; NS: Nerve sparing; HT: Hormone Treatment; QOL: Quality of life; F.up: Follow up; UCLA-PCI: University of California, Los Angeles, Prostate Cancer Index; UF: Urinary Function; SF: Sexual Function; UB: Urinary Bother; SB: Sexual Bother;

## Competing interests

The authors declare that they have no competing interests.

## Authors' contributions

MG, AS have made substantial contributions to conception and design. ML, AM, MAR, SS, VV, AR, CA, FV, METM were involved in the acquisition of data. LM and VV have made significant assistance in the interpretation of data. JLG has been involved in drafting the manuscript or revising it critically for important intellectual content. GC and MC have given final approval of the version to be published. Each author should have participated sufficiently in the work to take public responsibility for appropriate portions of the content.
